# Humanized anti-CD33 CAR-T cells and antibody-drug conjugates for targeted therapy in acute myeloid leukemia

**DOI:** 10.3389/fmed.2026.1691417

**Published:** 2026-01-26

**Authors:** Lijun Chen, Honghong Duan, Chunling Huang, Yajing Xu, Zitong Wang, Huibin Huang

**Affiliations:** 1 Department of Endocrinology, The Second Affiliated Hospital of Fujian Medical University, Quanzhou, Fujian, China; 2 Department of Obstetrics and Gynecology, The Second Affiliated Hospital of Fujian Medical University, Quanzhou, Fujian, China; 3 Department of Electrocardiogram, The Second Affiliated Hospital of Fujian Medical University, Quanzhou, Fujian, China; 4 The Second Clinical Medical College of Fujian Medical University, Quanzhou, China; 5 Johns Hopkins University, Baltimore, MD, United States

**Keywords:** AML, antibody-drug conjugates, car-t, CD33 antigen, humanized monoclonal antibodies

## Abstract

**Background:**

Acute myeloid leukemia (AML) is a malignant disorder originating from myeloid hematopoietic stem and progenitor cells. Despite the availability of current treatment options, a significant number of patients fail to achieve complete remission after initial chemotherapy. CD33, a transmembrane protein highly expressed on AML cells, serves as a promising therapeutic target. This study aimed to develop and evaluate chimeric antigen receptor T cells (CAR-T) and antibody-drug conjugates (ADC) based on humanized antibodies, specifically targeting CD33, to assess their potential efficacy against AML.

**Methods:**

Monoclonal antibodies specific to human CD33 were generated by immunizing mice and then humanized. These humanized antibodies were then used to construct CAR-T cells and ADCs, and their cytotoxic properties were evaluated both *in vitro* and *in vivo*. In the in vivo experiments, mice bearing Molm13-Luciferase tumor cells were assigned to different treatment groups and were administered with saline, Gemtuzumab-MMAE, or Clone3HM-MMAE.

**Results:**

The *in vitro* experiments revealed that several antibody clones, including Clone2HM, Clone3HM, Clone5HM, Clone6HM, and Clone7HM, displayed strong cytotoxic effects against Molm13-Luciferase tumor cells when conjugated with MMAE, outperforming the positive control antibody Gemtuzumab-MMAE. In the *in vivo* studies, mice treated with Clone3HM-MMAE showed a significant reduction in tumor signals, which nearly disappeared in the latter stages of the experiment. This led to a substantially longer survival time compared to other groups. Additionally, the body weight of mice in all treatment groups remained stable throughout the treatment period, indicating a favorable safety profile.

**Conclusion:**

The CAR-T cells and ADCs developed in this study, based on humanized antibodies, showed significant anti-tumor efficacy in the AML model. Clone3HM-MMAE, in particular, demonstrated excellent anti-tumor activity along with a strong safety profile. These results strongly support the further development of targeted therapeutic strategies for AML.

## 1 Introduction

Acute myeloid leukemia (AML) is a malignant disorder of the myeloid hematopoietic system originating from hematopoietic stem and progenitor cells. In the United States, the incidence rate of AML is approximately 4.3 cases per 100,000 individuals per year (1, 2). The disease is particularly prevalent in older adults, with incidence rates increasing with advancing age. New AML cases among adults aged 65–74 account for 25.1% of all diagnoses, while those among adults aged 75 and older account for 33.7%. Mortality rates also rise with age, reaching 43.7% in patients aged 75 and above. The five-year overall survival rate for AML remains below 30% (3).

Although chemotherapy and hematopoietic stem cell transplantation (HSCT) have improved outcomes for some AML patients, 10–40% of newly diagnosed patients fail to achieve complete remission (CR) after initial chemotherapy (4). This underscores the urgent need for more effective therapeutic strategies to address the challenges of AML.

Chimeric antigen receptor (CAR) T cells are engineered autologous T cells expressing recombinant receptors that recognize and target specific tumor antigens. The CAR structure comprises an antigen-binding domain and T-cell signaling components (5). CAR T cells offer several advantages, including MHC-independent antigen recognition, higher specificity than T-cell receptors (TCRs), the ability to be programmed to target diverse tumor antigens, enhanced proliferation and persistence, tunable cytotoxicity, and mechanisms to counter tumor escape. These features position CAR T cells as a promising option for cancer therapy (6, 7). Recent studies have emphasized the critical role of cancer-specific target antigens in optimizing CAR T cell therapy for hematological malignancies, highlighting how antigen selection influences efficacy, persistence, and resistance mechanisms in diseases like AML and B-cell lymphomas (8). This approach has shown substantial efficacy in malignant diseases, particularly B-cell hematologic malignancies and multiple myeloma (9–12). For instance, anti-CD19 CAR T cells have demonstrated potent and sustained antitumor effects in acute lymphoblastic leukemia (ALL) and were the first such therapy approved by the U.S. Food and Drug Administration (FDA) (13).

CD33 is a transmembrane protein primarily expressed on myeloid cells. It is highly expressed on AML progenitor cells but absent on normal hematopoietic stem cells. Current evidence indicates that CD33 is present on myeloblasts, monocytes, and other myeloid lineages, with limited expression in non-hematopoietic tissues. In myeloid leukemia cells, however, CD33 expression is substantially upregulated. As multipotent hematopoietic stem cells differentiate, CD34 expression decreases while CD33 expression emerges (14). This expression profile renders CD33 an attractive therapeutic target for myeloid leukemias.

Gemtuzumab ozogamicin (US7727968) is a humanized monoclonal IgG4 antibody targeting human CD33. When conjugated to a calicheamicin derivative, it binds to CD33-expressing leukemic blasts. Following internalization, the linker hydrolyzes, releasing the toxin, which binds to DNA and induces site-specific double-strand breaks, leading to cell death. CAR T cells engineered with this antibody exhibit potent cytotoxicity against CD33-positive myeloid leukemia cells (15). Furthermore, bispecific antibodies fusing anti-CD33 and anti-CD3 components can redirect T cells to tumor cells, eliciting robust cytotoxic responses against myeloid leukemia (16). These strategies broaden the scope of CD33-targeted therapies by engaging the immune system through diverse mechanisms.

Despite these advances, current CD33-targeted therapies face significant limitations that hinder their widespread clinical adoption. Gemtuzumab ozogamicin, while effective in select patient subsets, is associated with notable toxicities, including hepatotoxicity (such as veno-occlusive disease), prolonged myelosuppression, and infusion-related reactions, which contributed to its temporary market withdrawal in 2010 (17, 18). CD33-directed CAR T cells and bispecific antibodies often suffer from on-target/off-tumor effects due to CD33 expression on normal myeloid progenitors, leading to severe cytopenias and the potential need for bridging allogeneic transplantation (19). Additional challenges include antigen escape mechanisms, limited persistence in the immunosuppressive AML microenvironment, potential immunogenicity from non-fully humanized antibody components, and manufacturing complexities in pretreated patients (20). These drawbacks highlight the necessity for optimized next-generation agents with improved safety, specificity, and efficacy profiles.

To address these limitations, this study developed novel fully humanized anti-CD33 monoclonal antibodies designed to enhance affinity, reduce immunogenicity, and minimize off-target effects. Leveraging these antibodies, we constructed CAR T cells and antibody-drug conjugates (ADCs) to evaluate their preclinical efficacy and safety in AML models, with the goal of offering safer and more effective therapeutic options for patients facing this challenging disease.

## 2 Materials and methods

### 2.1 Antigen preparation

The sequence of the extracellular region of human CD33 (Asp 18 - His 259) was retrieved from the UniProt database (UniProt Accession# P20138). A His tag was attached to the C-terminus, and the codons were optimized for human codon usage. After gene synthesis, the gene was subcloned into the pcDNA3.4 eukaryotic expression vector. The sequence was confirmed through Sanger sequencing, and the plasmid was prepared for further applications. The modified expression vector was transiently transfected into 293F cells, the supernatant containing the expressed protein was collected, and the target protein was purified using nickel affinity chromatography. Protein purity was assessed by SDS-PAGE, and its concentration was determined using a NanoDrop 2000. The purified protein was used for immunization and subsequent antibody screening.

### 2.2 Construction of CHO-K1-CD33 cell line

Using the full-length amino acid sequence of human CD33 from the UniProt database, the gene was synthesized commercially and subsequently subcloned into the Lenti-CMV-puro lentiviral expression vector, thereby constructing the Lenti-CMV-CD33-puro overexpression vector. Following lentivirus packaging, 100 μL of the virus was added to CHO-K1 cells. After 48 h, 8 μg/mL puromycin was introduced to select for the CHO-K1-CD33 cell line. Once selection was complete, Flow cytometry analysis using a positive control antibody was conducted to confirm surface expression of human CD33.

### 2.3 Mouse immunization

All mice were maintained in a barrier system, where they were provided with sterilized pellet feed and autoclaved water. Five SPF-grade BALB/c mice were identified using ear tags. Each mouse received a subcutaneous injection of 100 μg of human CD33 protein, emulsified with Freund’s adjuvant, administered at multiple sites. Three immunizations were administered at 14-day intervals. Following the final immunization, peripheral blood was collected from the tail vein, and serum was isolated. The immunization titers were then evaluated using FACS.

### 2.4 Hybridoma fusion and monoclonal screening

The mouse with the highest immunization titer was selected for a three-day booster immunization. Under sterile conditions, the spleen was isolated, and a single-cell suspension of B cells was prepared. These B cells were then combined with SP2/0 myeloma cells at a 1:1 ratio, and cell fusion was performed using a BTX cell electrofusion system (21). Following electrofusion, cells were immediately resuspended in complete medium (DMEM, 20% FBS, and HAT) and transferred to 96-well plates for incubation. Approximately 10 days after fusion, the medium was replaced with HT medium. After 2 days, the supernatant was collected for ELISA to evaluate its binding to the human CD33 target antigen, and FACS was used to assess binding to CHO-K1-CD33 cells. Following each subcloning round, the ELISA and FACS assays were repeated according to the same protocol until monoclonal antibodies were successfully obtained.

### 2.5 Hybridoma sequencing

From the final selection, approximately 1 × 10^6^ hybridoma cells were collected and lysed using TRIzol reagent. Total RNA was extracted according to the manufacturer’s protocol, followed by reverse transcription into cDNA. The variable regions of the antibody heavy and light chains were amplified by PCR using hybridoma-specific sequencing primers. The resulting PCR fragments were cloned into a TA cloning vector via TA cloning. Positive clones were subjected to Sanger sequencing, and the obtained sequences were analyzed for antibody gene identification (22).

### 2.6 Construction and validation of chimeric antibody expression vector

Based on the variable region sequences obtained from hybridoma sequencing, expression vectors encoding chimeric antibodies were designed. The vectors were transiently transfected into serum-free cultured 293F cells. After 48 h, the culture supernatant containing the expressed antibody was collected, and antibody expression was confirmed by ELISA.

### 2.7 Humanization design of the antibody

**Figure 1:**
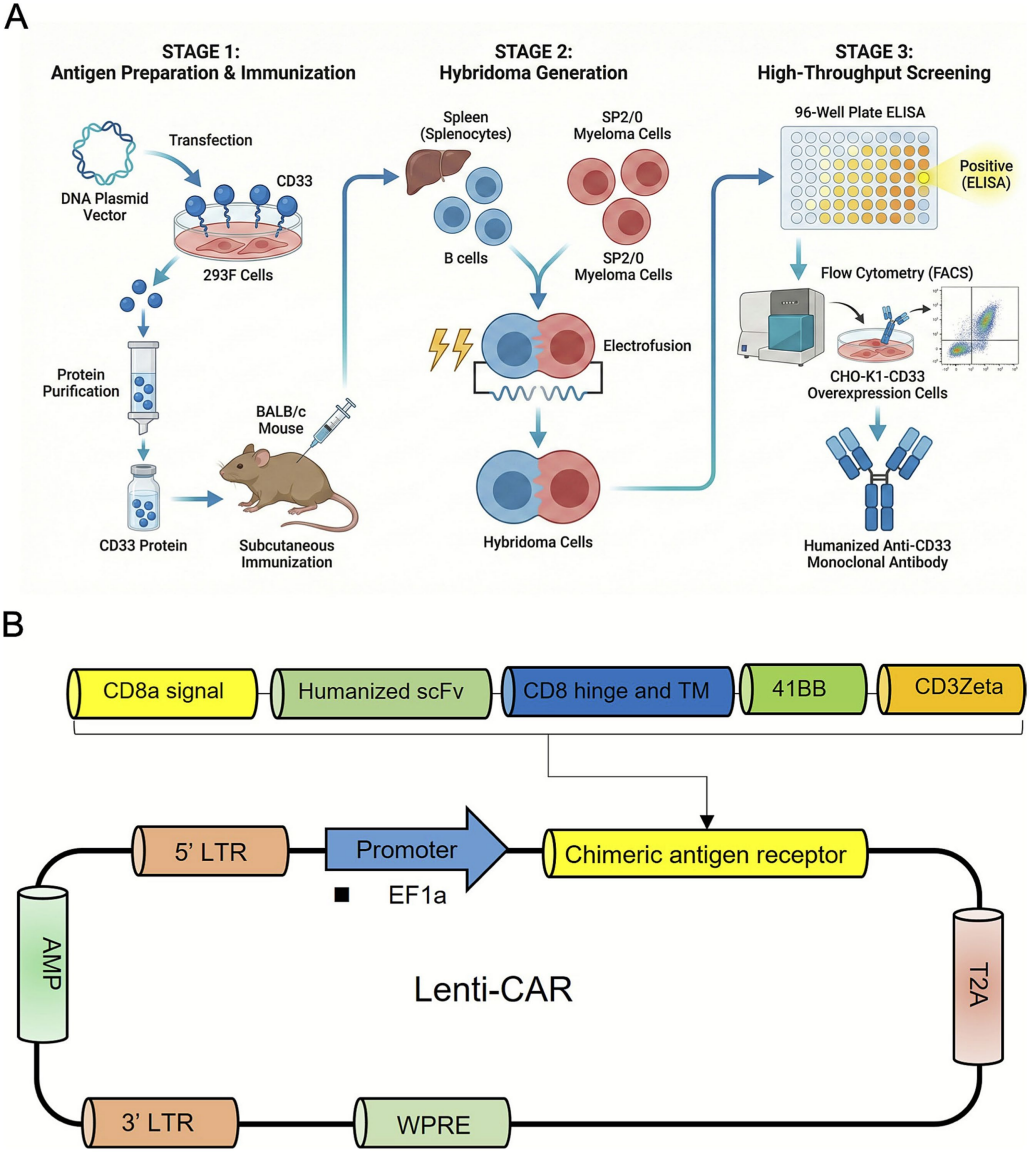
Schematic illustration of anti-CD33 monoclonal antibody generation and chimeric antigen receptor (CAR) construct design. **(A)** Workflow for the production and screening of anti-CD33 monoclonal antibodies. Stage 1: Antigen preparation and immunization. A DNA plasmid vector encoding CD33 is transfected into 293F cells for protein expression, followed by purification of the CD33 antigen, which is then administered subcutaneously to immunize Balb/c mice. Stage 2: Hybridoma generation. Splenocytes from immunized mice are fused with SP2/0 myeloma cells via electrofusion to produce hybridoma cells. Stage 3: High-throughput screening. Hybridoma supernatants are initially screened using enzyme-linked immunosorbent assay (ELISA) in 96-well plates, with positive clones further validated by flow cytometry (FACS) on CHO-K1 cells overexpressing CD33 to identify specific anti-CD33 antibodies. Panel A created with BioRender.com. **(B)** Schematic diagram of the lentiviral vector (Lenti-CAR) encoding the anti-CD33 CAR. The construct includes the 5′ long terminal repeat (LTR), ampicillin resistance gene (AMP), EF1α promoter driving expression of the chimeric antigen receptor composed of a CD8α signal peptide, humanized single-chain variable fragment (scFv), CD8 hinge and transmembrane (TM) domain, 4-1BB costimulatory domain, and CD3ζ signaling domain, followed by a T2A self-cleaving peptide, 3′ LTR, and woodchuck hepatitis virus posttranscriptional regulatory element (WPRE).

Using the amino acid sequences of the murine heavy and light chains, a humanization design was developed. The original complementarity-determining regions (CDRs) sequences were retained, while the human antibody templates for the heavy and light chains were selected based on the results of germline alignment and structural modeling. Back mutations were introduced into the framework regions after humanization to refine the candidate humanized antibody sequences. The genes for the humanized heavy and light chains were synthesized separately, with the heavy chain inserted into the pcDNA3.4-hIgG1 vector and the light chain into the pcDNA3.4-hIgKc vector. After confirming the accuracy of the sequences, the vectors were transiently transfected into 293F cells. The culture supernatant was collected, and the recombinant humanized antibody was purified using Protein A affinity chromatography. The binding of the candidate antibody to CHO-K1-CD33 cells overexpressing the target CD33 protein was then evaluated using FACS (Figure 1A).

### 2.8 Preparation of CAR-T cells

Based on the humanization screening, the following humanized clones were selected for CAR-T construction: Clone2HMCAR, Clone3HMCAR, Clone5HMCAR, Clone6HMCAR, Clone7HMCAR, Clone6-VL3VH1, Clone6-VL1VH2, and Clone2-VL3VH1. Lentiviral vectors encoding chimeric antigen receptors (CARs) incorporating 4-1BB and CD3ζ signaling domains were constructed for each clone. Recombinant lentiviruses were produced and used to transduce primary human T cells to generate CAR-T cells for functional evaluation (Figure 1B; Supplementary Data S1).

### 2.9 Lysis of target cells by CAR-T cells

First, Molm13-Luciferase target cells in the logarithmic growth phase were harvested and resuspended in complete culture medium (RPMI-1640 supplemented with 10% FBS) at a density of 2 × 10^5^ cells/mL. A total of 100 μL of cell suspension was seeded into each well of a 96-well plate. The outer wells of the plate were filled with 100 μL sterile phosphate-buffered saline (PBS) to minimize evaporation during incubation. The plate was incubated overnight at 37 °C in a humidified 5% CO₂ atmosphere.

On the following day, CAR-T cells were centrifuged, resuspended in fresh complete medium, and counted. The culture medium was gently aspirated from the target cell-seeded wells, and the adherent cells were washed once with pre-warmed PBS. CAR-T cells were then added to the wells at specified effector-to-target (E: T) ratios. For the maximum luminescence control (RLU_max), wells containing only target cells without CAR-T cells were included. The final volume in each well was adjusted to 200 μL with complete medium. The plate was incubated for 18 h at 37 °C under 5% CO₂.

After incubation, the plate was centrifuged briefly, and 100 μL of supernatant from each well was carefully removed and stored at −80 °C for subsequent cytokine analysis by enzyme-linked immunosorbent assay (ELISA). To assess cytotoxicity, 100 μL of Bright-Glo™ Luciferase Assay reagent (or equivalent) was added to each well according to the manufacturer’s instructions. Luminescence was measured using a microplate reader. The percentage of specific lysis was calculated as follows:


$$\text{Lysis}\%=(1-\frac{\mathrm{RL}U_{\text{Sample}}}{\mathrm{RL}U_{\max}})\times 100\%$$

RLU_sample represents the luminescence signal from wells containing both CAR-T cells and target cells, and RLU_max is the signal from wells containing target cells alone (maximum luminescence control).

### 2.10 *In vivo* efficacy validation of CAR-T cells

Fifteen 6- to 8-week-old female NSG mice (body weight 18–22 g) were used. After 1 week of acclimatization, each mouse was intravenously injected via the tail vein with 3.3 × 10^5^ Molm13-Luciferase (Molm13-Luc) cells in 200 μL PBS to establish a disseminated leukemia model. Seven days post-tumor inoculation (designated as Day 0), mice were randomly assigned into five treatment groups (n = 3 per group) based on body weight: (1) PBS vehicle control, (2) untransduced T cells (UTD), (3) Clone2HM-CAR T cells, (4) Clone6HM-CAR T cells, and (5) Gemtuzumab-CAR T cells (positive control). On Day 0, each mouse received a single intravenous injection of 1.5 × 10^7^ respective CAR-T cells or control T cells in 200 μL PBS. Tumor growth was monitored weekly via *in vivo* bioluminescence imaging (BLI) beginning on Day 7, with subsequent imaging sessions on Days 14, 21, and 28 post-treatment. Mice were euthanized when they reached predefined humane endpoints.

### 2.11 Cytotoxic effect of the candidate antibody conjugated with a toxin on target cells

The candidate humanized antibodies and the control antibody (Gemtuzumab) were conjugated to monomethyl auristatin E (MMAE) via a cleavable maleimide linker (Mal-PEG8-VC-PAB-MMAE) using a thiol-based conjugation strategy. Briefly, each antibody was partially reduced by incubation with a 6-fold molar excess of tris(2-carboxyethyl)phosphine (TCEP) at 25 °C for 6 h to generate reactive interchain cysteine thiols. Subsequently, a 10-fold molar excess of the linker-payload (Mal-PEG8-VC-PAB-MMAE) was added, and the conjugation reaction was allowed to proceed for 4 h at 25 °C. The reaction was quenched by adding a 20-fold molar excess of L-cysteine. To purify the ADC, the reaction mixture was subjected to Protein A affinity chromatography to remove excess unconjugated linker-payload and small-molecule reagents. The bound ADC was eluted using 0.1 M glycine-HCl buffer (pH 3.0) and immediately neutralized with 1 M Tris–HCl buffer (pH 9.0). The eluate was buffer-exchanged into PBS (pH 7.4) using a desalting column, sterile-filtered (0.22 μm), and concentrated. The final drug-to-antibody ratio (DAR) of each ADC was determined by hydrophobic interaction chromatography-high-performance liquid chromatography (HIC-HPLC).

### 2.12 *In vitro* cytotoxicity of the candidate antibody conjugated with MMAE against target cells

Molm13-Luciferase cells were taken during the logarithmic growth phase, and their density was adjusted to 2 × 10^5 cells/mL using complete medium. A 96-well plate was prepared by adding 100 μL of sterile water to the outer wells, while 100 μL of the cell suspension was added to the remaining wells. The plate was then incubated at 37 °C in a 5% CO2 atmosphere for 24 h.

Next, the conjugated candidate antibody and the positive control antibody were filtered through a 0.22 μm membrane for sterilization. The antibody concentration was adjusted to 20 μg/mL using complete medium. From the first gradient concentration stock solution, 240 μL (at 20 μg/mL) was diluted in 480 μL of medium. A threefold serial dilution was then performed to generate nine different concentrations. Subsequently, 100 μL of each antibody dilution was added to the corresponding wells.

After 120 h of incubation, cell viability was assessed using a luciferase-based assay. Briefly, 100 μL of D-luciferin potassium salt solution (150 μg/mL in PBS) was added to each well. The plate was gently mixed on an orbital shaker for 2 min and incubated at room temperature for 10 min to stabilize the luminescent signal. Subsequently, 100 μL of the mixture from each well was transferred to a white, opaque-walled 96-well plate, and luminescence was measured using a Tecan M1000Pro microplate reader.

### 2.13 *In vivo* cytotoxicity of the candidate antibody conjugated with MMAE against target cells

Molm13-Luc cells were thawed from liquid nitrogen and expanded in culture to reach the logarithmic growth phase. The cells were then harvested and resuspended in sterile PBS at a density of 5 × 10^6^ cells/mL. Female NSG mice (6–8 weeks old) were intravenously inoculated via the tail vein with 5 × 10^5^ Molm13-Luc cells in 100 μL PBS per mouse to establish a disseminated leukemia model.

Seven days post-tumor inoculation (Day 0 of treatment), baseline tumor burden was assessed by bioluminescence imaging (BLI). Briefly, mice were anesthetized and administered 200 μL of D-luciferin potassium salt (15 mg/mL in PBS) intraperitoneally. Imaging was performed 10–15 min post-injection using an IVIS® Spectrum system. Immediately after baseline imaging, mice were randomly assigned into three treatment groups (n = 6–8 per group) based on tumor signal intensity and body weight. Groups received a single intravenous injection of: (1) saline vehicle control, (2) Gemtuzumab-MMAE (5 mg/kg), or (3) Clone3HM-MMAE (5 mg/kg).

Tumor progression was monitored weekly by BLI as described above, on Days 7, 14, 21, and 28 post-treatment. Mice were euthanized when they reached predefined humane endpoints (e.g., >20% body weight loss, signs of distress, or hind-limb paralysis). Survival was recorded and analyzed.

### 2.14 Statistical analysis

SPSS 21.0 software was used for statistical analysis. Significance was determined by Student *t*-test or ANOVA. Results are expressed as mean ± SD. All reported *p*-values were two-tailed, and a value of *p* < 0.05 was considered statistically significant.

## 3 Results

### 3.1 FACS results of candidate clones binding to target protein

**Figure 2:**
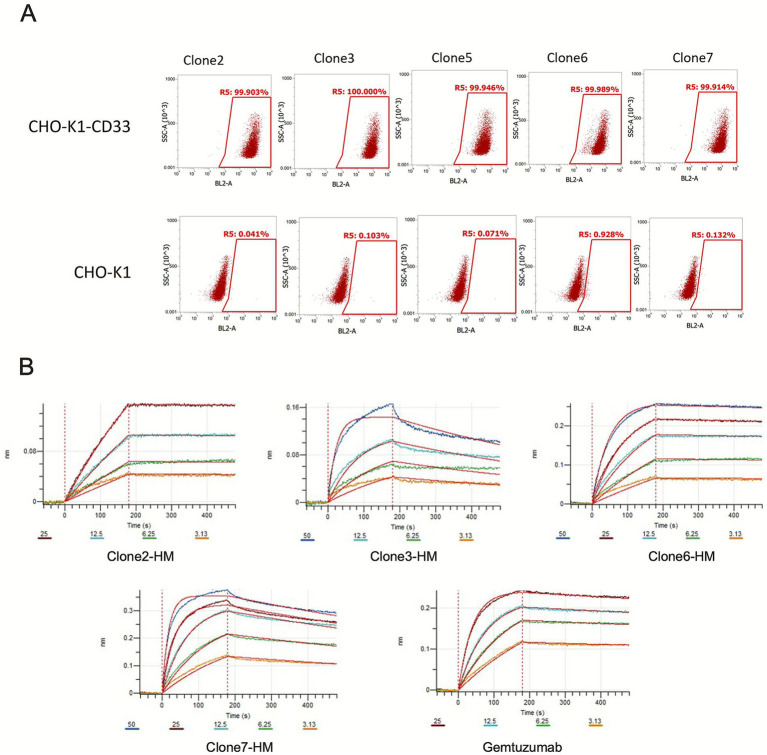
Characterization of anti-CD33 antibody binding specificity and affinity. **(A)** Flow cytometry analysis of candidate monoclonal antibody binding to CD33-expressing cells. Histograms depict the binding profiles of five murine-derived anti-CD33 monoclonal antibody clones (Clone 2, Clone 3, Clone 5, Clone 6, Clone 7) and their corresponding humanized variants (Clone2-HM, Clone3-HM, Clone6-HM, Clone7-HM). Stable CD33-overexpressing CHO-K1 cells (CHO-K1-CD33, top row) and wild-type CHO-K1 cells lacking CD33 expression (CHO-K1, bottom row) were stained with the respective primary antibodies followed by a fluorescently labeled secondary antibody. The X-axis (BL2-A channel) indicates fluorescence intensity corresponding to antibody binding, and the Y-axis (SSC-A) represents side scatter, a measure of cellular granularity. The percentage of positively stained cells (%RS, Region Statistics) is annotated within each gate. All clones and their humanized variants demonstrate strong, specific binding to CHO-K1-CD33 cells (>99.9% positivity for most), with negligible background binding to control CHO-K1 cells (<0.2% positivity, except Clone 7 at 0.92%), confirming target-specific recognition. Data are representative of at least two independent experiments. **(B)** Surface plasmon resonance (SPR) analysis for kinetic affinity determination of humanized antibodies. Affinity testing of humanized antibodies using the BIAcore T200 system. The sensorgrams show the binding response for each antibody at concentrations of 50 nM, 25 nM, 12.5 nM, 6.25 nM, and 3.13 nM. The x-axis represents time (seconds), and the y-axis represents response units (RU). The different colored curves correspond to the antibody concentrations. The data confirm that humanization preserved the nanomolar-range binding affinity of the parental antibodies.

Flow cytometry was performed to assess the binding specificity of various antibody clones (Clone2, Clone3, Clone5, Clone6, Clone7) to CD33-expressing CHO-K1 cells (CHO-K1-CD33) and non-expressing CHO-K1 cells. For CHO-K1-CD33 cells, all clones exhibited very high binding positivity, with the percentage in the R5 region approaching 100%. This indicates that the antibodies from these clones efficiently bind to CD33-expressing CHO-K1 cells. In contrast, for CHO-K1 cells lacking CD33 expression, the binding rates were very low, with the R5 region percentage ranging from 0.041 to 0.928%. This demonstrates minimal non-specific binding to CHO-K1 cells without CD33, indicating good specificity (Figure 2A).

### 3.2 Affinity testing of humanized antibodies

Affinity testing of various humanized antibody clones (Clone2-HM, Clone3-HM, Clone6-HM, Clone7-HM) and the control antibody Gemtuzumab showed that all clones exhibited good binding characteristics. Clone7-HM and Gemtuzumab demonstrated the highest *Kon* (association rate constant) and the lowest *Koff* (dissociation rate constant), indicating that their binding to the antigen is both rapid and stable, with strong affinity. Clone2-HM and Clone6-HM also displayed favorable association and dissociation rates, indicating strong affinity overall. Although Clone3-HM showed slightly weaker affinity, its KD value was still within an acceptable range, suggesting potential for therapeutic application. Overall, all tested clones demonstrated good affinity, especially Clone7-HM, which showed excellent binding stability (Figure 2B and Table 1).

**Table 1:** Affinity data of different humanized antibodies.

Clone ID	Kon	Koff	KD
Clone2-HM	1.069 × 10^5^	3.849 × 10^−5^	3.599 × 10^−10^
Clone3-HM	8.873 × 10^5^	1.311 × 10^−3^	1.478 × 10^−9^
Clone6-HM	4.402 × 10^5^	9.216 × 10^−5^	2.094 × 10^−10^
Clone7-HM	1.150 × 10^6^	7.709 × 10^−4^	6.702 × 10^−10^
Gemtuzumab	1.05 × 10^6^	2.215 × 10^−4^	2.110 × 10^−10^

Kon, Association Rate Constant; Koff, Dissociation Rate Constant; KD, Equilibrium Dissociation Constant.

### 3.3 Cytotoxicity of CAR-T cells constructed with candidate humanized antibodies against target cells

**Figure 3:**
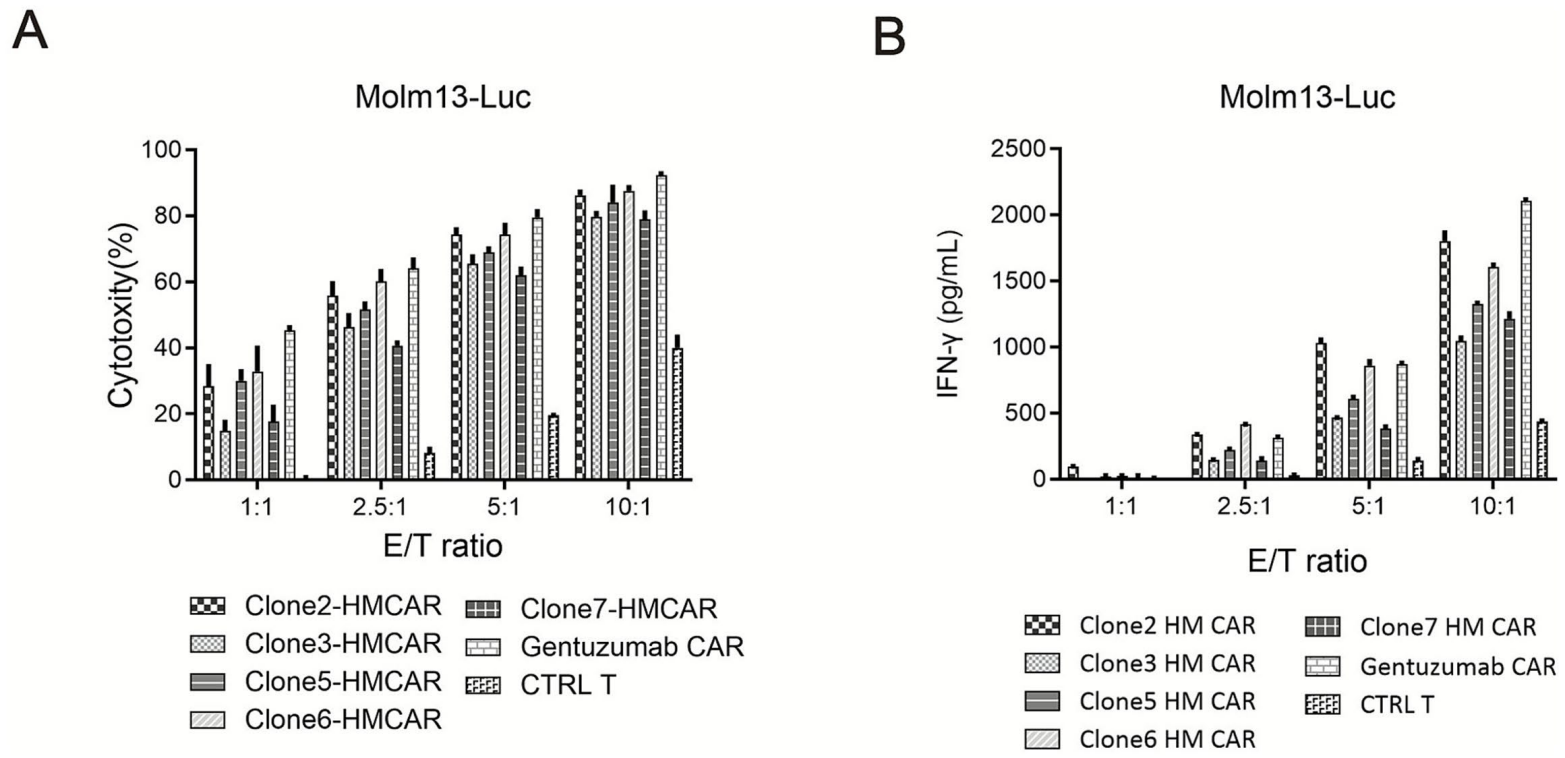
*In vitro* functional evaluation of CD33-targeting CAR-T cells bearing humanized antibody variants. **(A)** Cytotoxicity of anti-CD33 CAR-T cells against CD33-positive leukemia cells. The specific killing activity of primary human T cells transduced with CAR constructs encoding different humanized anti-CD33 scFv variants (Clone2-HMCAR, Clone3-HMCAR, Clone5-HMCAR, Clone6-HMCAR, Clone7-HMCAR) was assessed using a real-time luciferase-based cytotoxicity assay. Target CD33 + acute myeloid leukemia cell line Molm13, engineered to stably express luciferase (Molm13-Luc), was co-cultured with the respective CAR-T cells at effector-to-target (E/T) ratios of 1:1, 2.5:1, 5:1, and 10:1 for 24 h. A CAR construct incorporating the scFv from the clinically validated antibody Gemtuzumab (Gemtuzumab CAR) served as a positive control, while untransduced T cells (CTRL T) served as a negative control. Cytotoxicity (%) was calculated based on the reduction in luminescence signal relative to target cells alone. Data are presented as mean ± SD from three independent experiments performed in triplicate. All humanized CAR-T variants exhibited dose-dependent cytotoxicity, with Clone2-HMCAR and Clone3-HMCAR showing potent activity comparable to the Gemtuzumab CAR control at higher E/T ratios. **(B)** Antigen-specific IFN-*γ* secretion by anti-CD33 CAR-T cells upon target engagement. The functional cytokine response of the same panel of CAR-T cells was measured after stimulation with Molm13-Luc target cells. Following a 24-h co-culture at the indicated E/T ratios (1:1, 2.5:1, 5:1, 10:1), supernatant was harvested, and IFN-γ concentration was quantified by enzyme-linked immunosorbent assay (ELISA). Data are shown as mean ± SD from three independent experiments performed in duplicate. The Gemtuzumab CAR-T cells and untransduced T cells (CTRL T) served as positive and negative controls, respectively. IFN-γ secretion correlated with cytotoxic activity, with the most potent cytotoxic clones (Clone2-HMCAR, Clone3-HMCAR) also producing the highest levels of IFN-γ in an E/T ratio-dependent manner, confirming effective T-cell activation upon antigen recognition.

An *in vitro* co-culture system was established using Molm13-Luciferase as target cells and CAR-T cells expressing different humanized antibodies as effector cells, at the effector-to-target ratios shown in Figure 3A. The results showed that CAR-T cells constructed with the candidate antibodies exhibited strong cytotoxicity against Molm13-Luciferase cells compared to control T cells. The cytotoxicity was consistent with that of CAR-T cells constructed using the positive control antibody Gemtuzumab.

### 3.4 CAR-T cell cytokine secretion

An *in vitro* co-culture system was established using Molm13-Luc as target cells and the prepared CAR-T cells as effector cells, at the effector-to-target ratios shown in Figure 3B. The results showed that, compared to control T cells, CAR-T cells significantly increased IFN-*γ* secretion after stimulation with Molm13-Luc cells, consistent with those constructed using the positive control antibody Gemtuzumab (Figure 3B).

### 3.5 *In vivo* efficacy testing of CAR-T cells

**Figure 4:**
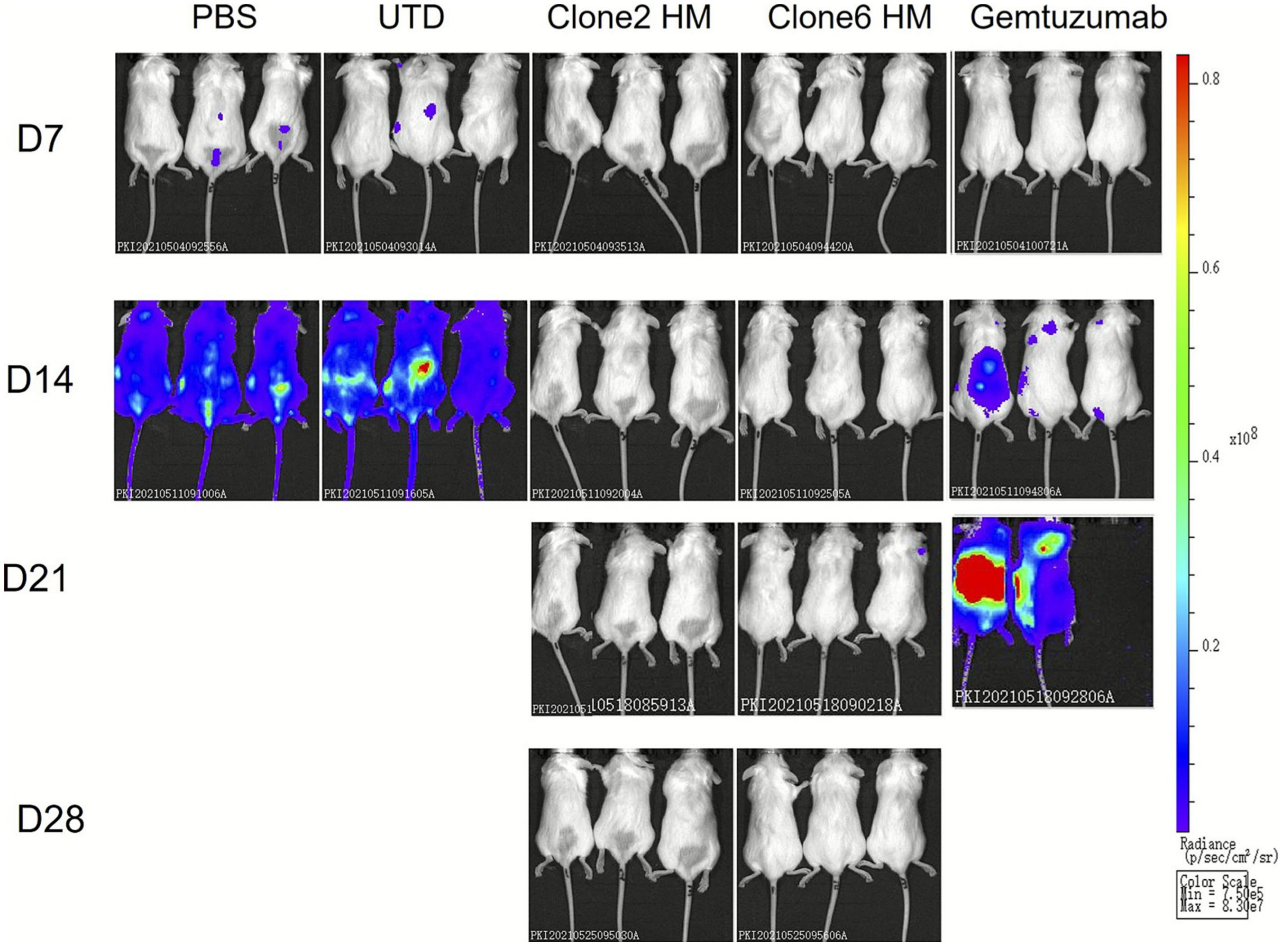
Inhibition of Molm13-Luc Tumor Growth by CAR-T Cells with Different Treatments. This figure shows the *in vivo* imaging results of mice treated with PBS, UTD, Clone2HM CAR-T, Clone6HM CAR-T, and Gemtuzumab CAR-T on day 7 (D7), day 14 (D14), day 21 (D21), and day 28 (D28). The colors represent the intensity of tumor activity. In the Clone2-HM CAR-T and Clone6-HM CAR-T treatment groups, tumor signals in the mice remained low throughout the observation period, demonstrating good anti-tumor effects.

Molm13-Luc cells, a CD33-positive tumor cell line in logarithmic growth phase, were resuspended in PBS. Each mouse received 200 μL of the cell suspension, containing 3.3 × 10^5 tumor cells, and administered via tail vein injection. The mice were randomly divided into five groups based on body weight: PBS, UTD, Clone2-HM, Clone6-HM, and Gemtuzumab. On day 0 (D0), CAR-T cells or control T cells were administered via tail vein injection, with each mouse receiving 1.5 × 10^7 CAR-T cells. The first *in vivo* imaging was conducted on day 7 (D7), followed by imaging every 7 days for a total of four imaging sessions. In the PBS and UTD groups, tumor signals in the mice persisted and gradually increased at different time points during the experiment, indicating continuous tumor growth. In the Clone2-HM CAR-T and Clone6-HM CAR-T treatment groups, tumor signals in the mice remained low throughout the observation period, demonstrating good anti-tumor effects. In the Gemtuzumab CAR-T treatment group, the tumor signals decreased on day 14, showing initial tumor suppression. However, by day 21 and day 28, the tumor signals increased again, suggesting tumor recurrence or regrowth in the later stages of treatment. The imaging results demonstrated that the CAR-T cells constructed with the humanized antibodies from this invention significantly inhibited tumor cell line growth in mice (Figure 4).

### 3.6 *In vitro* cytotoxicity of antibody-toxin conjugates against target cells

**Figure 5:**
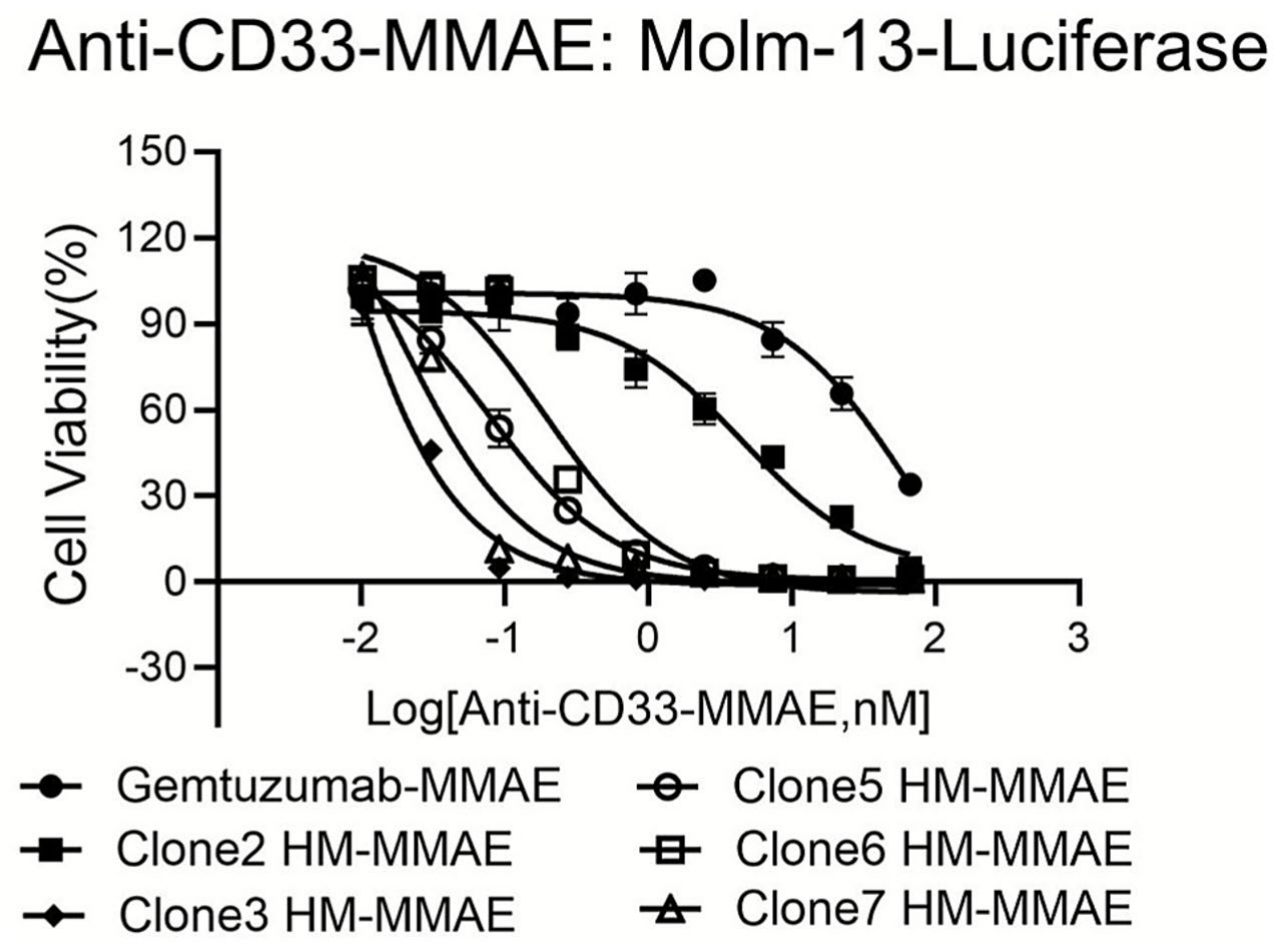
Cytotoxic activity of anti-CD33 antibody-drug conjugates against CD33-positive leukemia cells *in vitro*. The cytotoxic efficacy of humanized anti-CD33 monoclonal antibodies conjugated to the microtubule-disrupting agent monomethyl auristatin E (MMAE) was assessed in vitro against the CD33-positive acute myeloid leukemia cell line Molm13, engineered to express luciferase (Molm13-Luc). Cells were treated for 96 h with a serial dilution of the clinical benchmark ADC, Gemtuzumab ozogamicin analog (Gemtuzumab-MMAE), and five candidate humanized antibody-MMAE conjugates (Clone2HM-MMAE, Clone3HM-MMAE, Clone5HM-MMAE, Clone6 HM-MMAE, Clone7HM-MMAE). Cell viability was determined using a luminescence-based cell viability assay and is expressed as a percentage relative to untreated control cells. Data points represent the mean ± SEM of at least three independent experiments, each performed in triplicate. The half-maximal inhibitory concentration (IC50) was calculated from non-linear regression analysis of the dose–response curves. All candidate humanized ADCs demonstrated potent, dose-dependent cytotoxicity against Molm13-Luc cells, with several clones (notably Clone2HM-MMAE and Clone3HM-MMAE) exhibiting sub-nanomolar IC50 values comparable to or surpassing the potency of the Gemtuzumab-MMAE control.

All candidate antibodies (Clone2-HM-MMAE, etc.) conjugated with MMAE exhibited stronger cytotoxic effects on Molm13-Luciferase cells compared to the positive control Gemtuzumab-MMAE. This is especially evident at higher concentrations, where cell viability was lower, indicating enhanced cytotoxicity (Figure 5). Based on comprehensive profiling of binding kinetics, *in vitro* potency, and developability attributes, Clone3HM-MMAE was selected as the lead candidate for *in vivo* evaluation. It exhibited sub-nanomolar affinity (KD = 1.48 nM), rapid association kinetics (Kon = 8.87 × 10^5^ M^−1^ s^−1^), and potent cytotoxicity (IC₅₀ < 0.5 nM) comparable to Clone2HM-MMAE. These characteristics supported its advancement into preclinical efficacy studies.

### 3.7 *In vivo* cytotoxic effects of antibody-toxin conjugates on target cells

**Figure 6:**
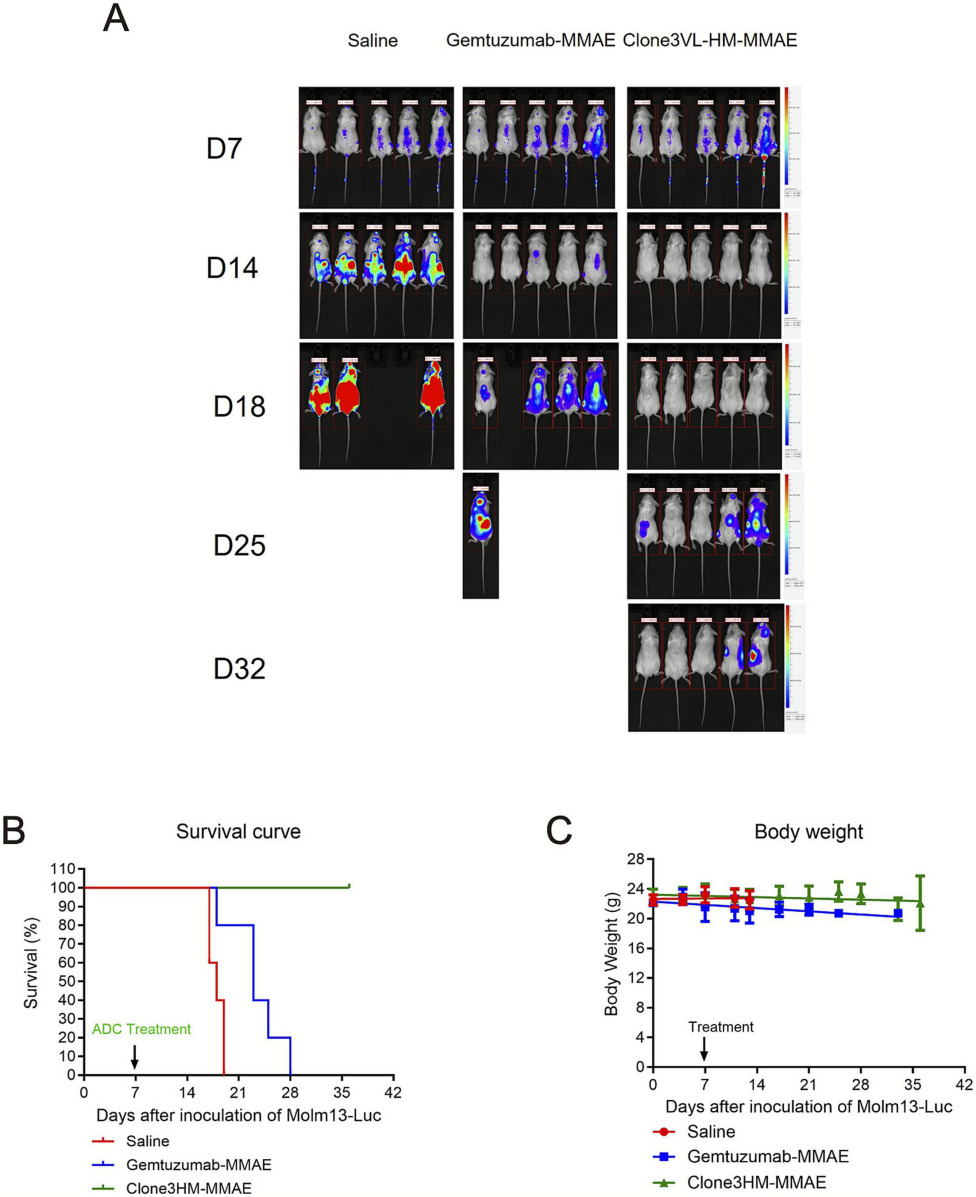
*In vivo* antitumor efficacy, survival benefit, and systemic toxicity assessment of anti-CD33-MMAE antibody-drug conjugates in a disseminated leukemia model. **(A)** Longitudinal bioluminescence imaging (BLI) of tumor progression following ADC treatment. NSG mice were intravenously inoculated with Molm13 cells stably expressing firefly luciferase (Molm13-Luc). On day 7 post-inoculation, mice were randomized into three treatment groups and received intravenous administration of: (1) saline vehicle control, (2) the clinical benchmark ADC Gemtuzumab ozogamicin analog (Gemtuzumab-MMAE, 3 mg/kg), or (3) the candidate humanized ADC Clone3HM-MMAE (3 mg/kg). Total tumor burden was monitored non-invasively via BLI at baseline (day 7) and subsequent time points (days 14, 18, and 25 post-inoculation). Representative pseudocolor BLI images overlaid on grayscale photographs are shown. The color scale indicates photon flux (photons/s/cm^2^/sr), with red representing high tumor burden. Mice treated with Clone3HM-MMAE showed rapid and near-complete suppression of BLI signal by day 14, which was sustained through day 25, demonstrating profound antitumor activity. Gemtuzumab-MMAE treatment also reduced tumor burden but with less complete suppression and earlier signal recurrence compared to Clone3HM-MMAE. Saline-treated mice exhibited progressive disease with exponentially increasing BLI signal. **(B)** Kaplan–Meier survival analysis. The survival curve illustrates the percentage of surviving mice in each group over time (days post tumor inoculation). Treatment with Clone3HM-MMAE resulted in a significant extension of median survival compared to the saline control group (*p* < 0.0001 by log-rank test) and showed a trend toward improved survival over the Gemtuzumab-MMAE group. **(C)** Body weight change as a measure of systemic toxicity. Mouse body weight was recorded every 2–3 days throughout the study period and is presented as mean body weight (grams) ± SEM for each group over time. Body weight loss >20% from baseline was considered a severe adverse event and a criterion for euthanasia. Mice treated with Clone3HM-MMAE maintained stable body weight throughout the treatment and follow-up period, comparable to the saline control group, indicating minimal treatment-related systemic toxicity at the administered dose. The Gemtuzumab-MMAE group exhibited a transient, modest weight loss (≤10%) following the first dose but recovered to baseline by the end of the treatment cycle.

The candidate humanized antibody Clone3-HM conjugated with a toxin, along with the positive control antibody (Gemtuzumab), effectively inhibited the proliferation of Molm13-Luciferase tumor cells in mice. The cytotoxic activity against the target cells was stronger than that of the positive control antibody Gemtuzumab, resulting in longer survival times for the mice. Additionally, there was no significant weight loss observed in the mice throughout the treatment period (Figure 6).

## 4 Discussion

The present study reports a panel of fully humanized anti-CD33 monoclonal antibodies generated through hybridoma technology followed by CDR-grafting. To comprehensively evaluate their therapeutic potential, we deployed these antibodies across two major platforms: as 4-1BB/CD3ζ second-generation CAR-T cells and as site-specifically conjugated MMAE-ADCs (average DAR ≈ 4). In the aggressive MOLM13-Luc xenograft model, lead clones—Clone3HM (ADC format) and Clone2HM/Clone6HM (CAR-T format)—displayed markedly superior anti-leukemic activity relative to benchmarks derived from gemtuzumab ozogamicin.

The superiority of Clone3HM-MMAE over gemtuzumab-MMAE is noteworthy. Despite a modestly higher equilibrium dissociation constant (KD 1.48 nM versus 0.21 nM for gemtuzumab), Clone3HM-MMAE achieved near-complete tumor eradication at 5 mg/kg and significantly prolonged survival without body-weight loss or other overt toxicity. This advantage probably derives from (i) enhanced internalization/lysosomal trafficking associated with the novel epitope, (ii) intrinsically higher potency of auristatin payloads compared with calicheamicin against CD33-expressing cells (23), and (iii) superior plasma stability and bystander activity of the Val-Cit-PAB-MMAE linker, as observed in contemporary CD33 and multispecific ADC platforms (24, 25). Preclinical data with other non-calicheamicin CD33 ADCs (e.g., GLK-33 lintuzumab–auristatin, BL-M11D1 exatecan derivative, TRX-214-1002 novel payload) similarly demonstrate widened therapeutic windows (23, 26, 27).

Although Clone3-HM exhibited the highest KD value (1.48 nM) among the panel—approximately 7-fold weaker than gemtuzumab (0.211 nM)—it demonstrated the most potent *in vivo* anti-tumor efficacy when conjugated to MMAE. This observation aligns with contemporary ADC optimization principles, wherein moderately reduced affinity (1–10 nM) coupled with exceptionally high association rates (kon > 8 × 10^5^ M^−1^ s^−1^) promotes superior tumor penetration, rapid internalization, and enhanced bystander killing, particularly when paired with cleavable, potent payloads such as MMAE (28, 29).

In the CAR-T arm, Clone2HM and Clone6HM CAR-T cells maintained undetectable bioluminescence through day 28, whereas gemtuzumab-based CAR-T exhibited late regrowth (Figure 4). This durable control occurred despite equivalent *in vitro* cytolysis and IFN-*γ* release, implying improved *in vivo* persistence and/or reduced exhaustion. Published clinical experience with lintuzumab-, hu195-, or affinity-optimized CD33 CAR-T constructs has consistently revealed transient responses and frequent CD33low/negative relapse unless rapidly bridged to allogeneic HSCT (30–33). The absence of late relapse in our lead CAR-T cohorts therefore constitutes a meaningful preclinical advance.

Shared limitations of all CD33-directed therapies persist. The NSG xenograft platform lacks human CD33-expressing normal myeloid progenitors and thus cannot predict clinical on-target/off-tumor myelotoxicity. Phase I/II trials of autologous or off-the-shelf CD33 CAR-T cells uniformly report prolonged grade 3–4 cytopenias lasting 4–12 weeks, frequently requiring rescue transplantation (33). Emerging CD33 CAR-NK platforms appear to mitigate hematopoietic toxicity while preserving efficacy (34).

As of November 2025, despite the approval of at least 12 new drugs or combination regimens for AML since 2017—a transformative period that has dramatically reshaped diagnosis, risk-stratification, measurable residual disease monitoring, and risk-adapted therapy—gemtuzumab ozogamicin remains the only CD33-directed agent approved for clinical use (35). This striking therapeutic gap, emphatically highlighted in the most authoritative 2025 annual clinical update on AML management, underscores the persistent unmet need for next-generation CD33-targeted approaches capable of overcoming the limitations of calicheamicin-based payload toxicity, suboptimal internalization kinetics, and vulnerability to CD33 splice-variant escape (35). The preclinical profile of Clone3HM-MMAE and Clone2HM/Clone6HM CAR-T cells—superior potency, durable disease control, preserved activity against the common CD33 splice variant, and favorable safety in xenograft models—strongly supports rapid IND-enabling development. Priority next steps should include (i) human CD33 knock-in or non-human primate toxicology studies, (ii) validation against primary CD33low and splice-variant patient samples, and (iii) rational combination strategies with hypomethylating agents or allogeneic HSCT bridging, as currently explored with VCAR33ALLO, DARIC33, and emerging CD33 ADCs (NCT05984199, NCT06315309) (28, 31).

## Data Availability

The raw data supporting the conclusions of this article will be made available by the authors, without undue reservation.

## Supplementary material

Supplementary Data S1 CAR Amino Acid Sequences.
