# Supplementary material for: Humanized anti-CD33 CAR-T cells and antibody-drug conjugates for targeted therapy in acute myeloid leukemia
Source: Front Med (Lausanne). 2026 Jan 26;13:1691417. doi: 10.3389/fmed.2026.1691417 (PMC12884322; doi:10.3389/fmed.2026.1691417)
Supplement: Supplementary Data S1 — CAR Amino Acid Sequences. [file Table_1.DOCX]

1. **Structure：**

**>CD8 signal peptide**

MALPVTALLLPLALLLHAARP

**>CD8 Hinge+transmembrane domain**

TTTPAPRPPTPAPTIASQPLSLRPEACRPAAGGAVHTRGLDFACDIYIWAPLAGTCGVLLLSLVITLYC

**>4-1BB signaling domain**

KRGRKKLLYIFKQPFMRPVQTTQEEDGCSCRFPEEEEGGCEL

**>CD3Zeta signaling domain**

RVKFSRSADAPAYKQGQNQLYNELNLGRREEYDVLDKRRGRDPEMGGKPRRKNPQEGLYNELQKDKMAEAYSEIGMKGERRRGKGHDGLYQGLSTATKDTYDALHMQALPPR

1. **scFv**

>Clone2

DIVMTQSPDSLAVSLGERATINCKASQNVNYYVAWYQQKPGQSPKLLIYYASSRYAGVPDRFTGSGYGTDFTLTISSLQAEDVAVYFCQQDYNSPYTFGGGTKLELKGGGGSGGGGSGGGGSQLQLVQSGAEVKKPGASVKVSCKASGYAFTNYLIEWVRQAPGQGLEWIGVINPGSGDTNYNEKFQGRVTLTADKSISTAYMELSRLRSDDTAVYFCARSPNYYGSSYFDYWGQGTLVTVSS

> Clone3

DIQMTQSPSSLSASVGDRVTITCKASQDINKYIAWYQHKPGKGPELLIYYTSTLHPGVPSRFSGSGSGRDYTLTISSLQPEDFATYYCLHYDNLLTFGGGTKVEIKGGGGSGGGGSGGGGSQVQLVQSGAEVKKPGASVKVSCKASGYTFTRYWMYWVRQAPGQGLEWIGEINPSNGQTNYAQKFQGRATLTVDKSTSTAYMELSSLRSEDTAVYYCARWHYGLDYWGQGTTVTVSS

>Clone5

DIQMTQSPSSLSASVGDRVTITCKANQDINQYIAWYQQKPGKGPKLLIYYTSLFQPGVPSRFSGSGSGRDYTLTISSLQPEDFATYYCLHYGNLLWTFGGGTKVEIKGGGGSGGGGSGGGGSQVTLKESGPTLVKPTQTLTLTCTFSGFSLNTSGLGVGWSRQPPGKALEWLALIWWDDIKYYSPSLKSRLTITKDTSKNQVVLTMTNMDPVDTATYYCARRGHNNAMDYWGQGTLVTVSS

>Clone6

DIQMTQSPSSLSASVGDRVTITCQASQDISNYLNWYQQKPGGAVKLLIYYTSRLHTGVPSRFSGSGSGTDYTFTISSLQPEDIATYYCQQGDTLPWTFGGGTKVEIKGGGGSGGGGSGGGGSEVKLVESGGGLVQPGGSLRLSCAASGFTFSNYAMSWVRQAPGKGLEWVASISSGGDTYYPDSVKGRFTISRDNSKNTLYLQMNSLRAEDTAVYYCVRGEANWDYFDYWGQGTLVTVSS

>Clone7

DIQMTQSPSSLSASVGDRVTITCKSSQDINKYIAWYQHKPGKGPKLLIRYTSILQPGVPSRFSGSGSGRDYTLTISSLQPEDFATYYCLHYENLLTFGGGTKVEIKGGGGSGGGGSGGGGSQVQLVQSGAEVKKPGASVKVSCKASGYTFTRYWIYWARQAPGQGLEWIGEIKPSDGRTNYAQKFQGRATLTVDESTSTVYMELSSLRSEDTAVYYCARWHYGLDYWGQGTTVTVSS
